# Design, Preparation and Thermal Characterization of Polystyrene Composites Reinforced with Novel Three-Cages POSS Molecules

**DOI:** 10.3390/molecules25132967

**Published:** 2020-06-28

**Authors:** Ignazio Blanco, Francesco Agatino Bottino, Gianluca Cicala, Giulia Ognibene, Claudio Tosto

**Affiliations:** 1 Department of Civil Engineering and Architecture (DICAr), University of Catania an UdR-Catania Consorzio INSTM, Viale Andrea Doria 6, 95125 Catania, Italy; gcicala@unict.it (G.C.); giuliaognibene@live.com (G.O.); claudio.tosto@unict.it (C.T.); 2 Department of Chemical Sciences (DSC), University of Catania, Viale Andrea Doria, 6 95125 Catania, Italy; fbottino@dii.unict.it

**Keywords:** polyhedral oligomeric silsesquioxanes, POSS, composites, thermal stability, polystyrene

## Abstract

Novel polystyrene (PS)/polyhedral oligomeric silsequioxanes (POSSs) nanocomposites were designed and prepared by in situ polymerization, using, for the first time, three-cage POSS molecules. The synthesized compounds were first characterized by Fourier transform infrared spectroscopy (FTIR) and ^1^H NMR spectroscopy to verify the obtaining of the designed products before their thermal performance was evaluated and compared with those of pristine PS and the corresponding single-cage POSSs nanocomposites. The thermal behaviour was checked by the means of the differential scanning calorimetry (DSC) and thermogravimetric analysis (TGA). Scanning electron microscopy (SEM) was also used to confirm the hypothesis about the dispersion/aggregation of the POSS molecules into the polymer matrix. The parameters chosen to evaluate the thermal stability of the investigated compounds, namely temperature at 5% of mass loss (*T*_5%_) and solid residue at 700 °C, showed a significant increase in the stability of the polymers reinforced with the three-cages POSS, in comparison to both PS and single-cage POSS reinforced PSs, which therefore turn out to be promising molecular fillers for nanocomposite production.

## 1. Introduction

**Figure 1:**
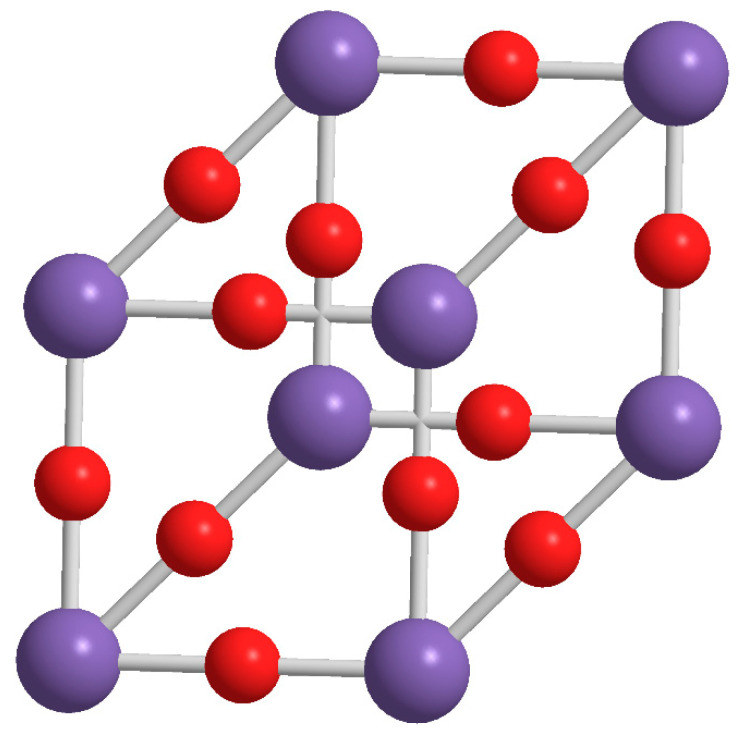
Molecular structure of single-cage POSS molecule.

Polystyrene (PS) is one of the oldest polymers, it was identified by Bonastre in the 1831 by the distillation of Storax obtained from the Turkish sweet gum trees, and distilled first time in 1939 by Simon as an oily liquid named styrol, then converted into a jelly product, the styroloxide. Industrially, polystyrene was manufactured starting from 1869 by Berthelot and the patents were finally granted to F.E. Matthew [1]. Then, in the last century, the academic and industrial activities regarding PS were continuously increasing due to its versatile applications and utility in various sectors. In particular, due to its ability to polymerize easily, PS is widely used in domestic, medical, and automotive applications [2,3,4]. The main application of PS is in packaging and building sectors, firstly as insulating material and in the last years for enhancing the design and structural integrity of the building [5]. Since PS is brittle and soften at relatively low temperature, the need of making composites for achieving the characteristics requested for a specific usage. Among the different PS composites [6,7,8,9], in the last two decades, increasing attention has been paid to those designed and realized by using polyhedral oligomeric silsesquioxanes (POSSs) [10,11,12]. Historically a lot of studies and application focused on those materials characterized by the R_2_SiO unit, inorganic silica and/or organic silicones, but more recently silsesquioxanes bearing general composition of RSiO_3/2_ gained growing interest [13]. This particular unit is due to the fact that each silicon atom is bonded to one-and-a-half oxygen and to a hydrocarbon by a condensation reaction. POSS molecules differ from other types of nanofillers, such as organoclay [14], carbon nanotube [15], nanofibers [16,17] because they can be dispersed in the polymer matrix at the level of individual molecules, being molecules instead of filler [18], therefore at a size much lower than the average dimension of conventional fillers [19]. Thus, among nanoreinforcements, functionalized organic/inorganic POSSs are considered unique nanobuilding blocks that can be used to create a wide variety of hybrid and composite materials, where precise control of nanostructures and properties is required [20]. Since 2012, the effect of POSSs incorporation in PS matrix by polymerization in situ was investigated by our research group at the University of Catania. Single-cage POSS molecules (Figure 1) with alkyl (having different chain length) or aryl substituents were firstly designed and studied [21,22], observing the effects of the substituents nature (alkyl or aryl) on the ability of the molecules to be dispersed in the matrix and thus on the properties of the corresponding nanocomposites [23,24].

**Figure 2:**
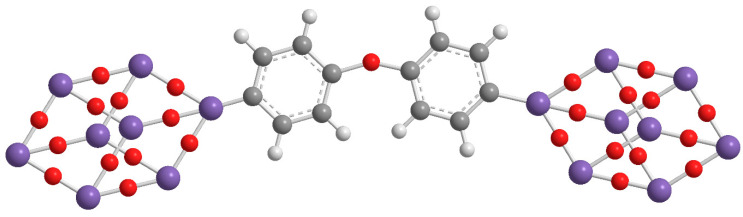
Molecular structure of double-cages POSS molecule.

During the following stage of the research, we focused our studies on the design, preparation and characterization of double-cage POSSs (dumbbell shaped POSS) [25] aiming at observing if the presence of a double cage (Figure 2) enhancing the performance of the POSSs into the polymer matrix. 

**Figure 3:**
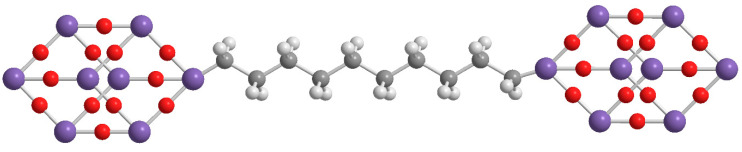
Molecular structure of aliphatic dumbbell shaped POSS.

If on the one hand the dumbbell shaped POSSs showed a greater resistance to the degradation with respect to the single cage POSSs, on the other hand we found a greater tendency to the aggregation phenomena, due to an increase in the symmetry of these double-cage nanomolecules [26]. This problem was partially solved by designing dumbbell shaped POSSs with an aliphatic bridge (Figure 3), instead of the aromatic one, which allowed greater flexibility and consequently a better dispersion of the nanomolecules into the matrix [18,27]. 

**Figure 4:**
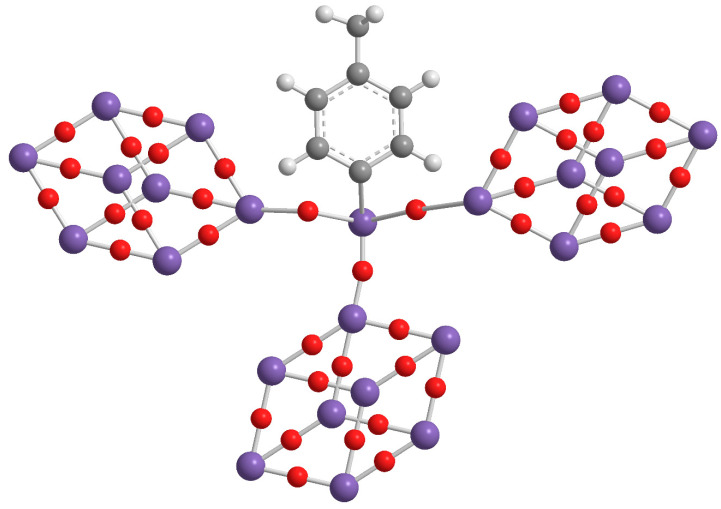
Molecular structure of three-cages POSS molecule.

The third level of our research was reached with the design and synthesis of a novel, and unique at moment, three-cages POSS (Figure 4) showing excellent performance [28].

In the present work we studied the effect of the incorporation of this novel POSS molecule into polystyrene matrix and we compared the performance of the obtained nanocomposites with those investigated in the past with single and double cages. Considering that the final properties of these materials are strictly depending on the morphology generated during their preparation, we tried to coupling spectroscopy study with thermal investigation in order to correlate the ability of POSSs to be dispersed at nanometric level with their thermal response.

Thus, we would to verify the possibility to prepare, by in situ polymerization of styrene, PS nanocomposites with about 5% *w*/*w* of three cages POSSs, functionalized with isobutyl or cyclopentyl peripheral groups. The POSS amount, dispersed into the matrix, was chosen on the basis of our previous investigations and literature evidences that suggested this concentration as the best one [18,29].

The synthesized POSS/PS nanocomposites were first spectroscopically characterized (^1^H NMR, SEM) and then their thermal performance was checked through the determination of the temperature at 5% mass loss (*T*_5__%_) and solid residue at 700 °C in inert and oxidative environment. The resistance to the thermal and thermooxidative degradation was evaluated by the means of thermogravimetric analysis (TGA), and by the solid residues after degradation by FTIR spectroscopy.

## 2. Results and Discussion

The designed nanocomposites (Table 1) were prepared starting from styrene and carrying out an in-situ polymerization by adding about 5% of POSS as reported in the experimental procedure. Once the PS/POSS nanocomposites were prepared, and named as shown in the Table 1, we proceed with the spectroscopic characterization aiming at establishing the actual presence of the silicon based molecular reinforcement in the polymeric matrix.

**Figure 5:**
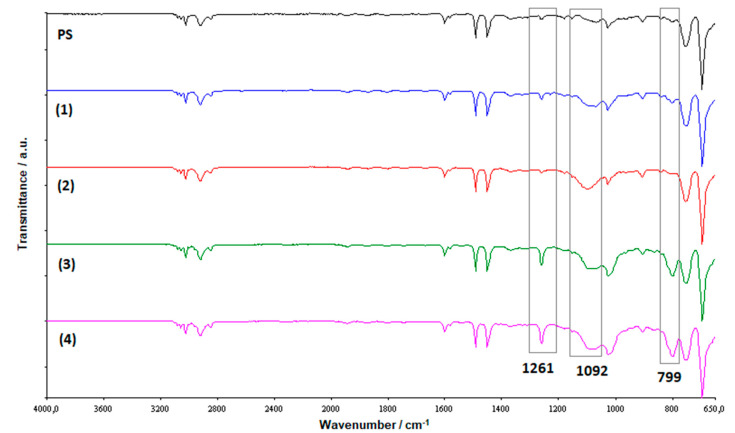
FTIR spectra of PS and prepared nanocomposites.

The nanocomposites where thus subjected to FT-IR measurements (Figure 5) that shown for samples **1**–**4**, with respect to the spectrum of pristine PS, the broad band at about 1090 cm^−1^ related to the asymmetric Si-O-Si stretching, belonging to the strong cage of the POSS [30,31]. This band that does not vary during the polymerization [28], because the cages of POSS do not break in the reaction [32], confirming, at least qualitatively, the presence of our POSS molecules in the polystyrene composites. The different molecular structures of the POSSs used as filler is highlighted by the increasing of the band at about 1260 cm^−1^ passing from samples **1** and **2** to samples **3** and **4**. This band, more intense for samples **3** and **4**, is attributable to Si-CH_2_ bonds [33], and the intensity increasing is due to the presence, in these samples, of filler molecules with three silicon cages, differently than in the samples **1** and **2** for which only one silicon cage is present in the POSSs used for reinforcing the matrix. The FT-IR allowed us a further discrimination among the different nanocomposites because of the band at 799 cm^−1^, presents only for samples **3** and **4**, which is attributable to the methyl phenyl group [34] linked to the silicon atom that holds the three silicon cages together.

To be sure that the designed molecular structure of the nanocomposites was effectively obtained after the polymerization and to check their actual POSSs content, ^1^H NMR analysis was carried out on the synthesized samples. Data collected from ^1^H NMR allowed us to determine the following percentage 7.6%, 7.1%, 6.2%, and 7.5% for the compounds **1**, **2**, **3**, and **4**, respectively. The determined, considering the ratio of hydrogen atoms of nanoparticles and those of polymer in the ^1^H NMR spectra, POSSs percentages in the various PS nanocomposites, were slightly higher than those of starting mixtures. This shift upwards of the POSSs content was attributed, in agreement with literature [35], to the formation, during the in-situ polymerization, of PS oligomers [36,37] that were soluble in methanol. 

**Figure 6:**
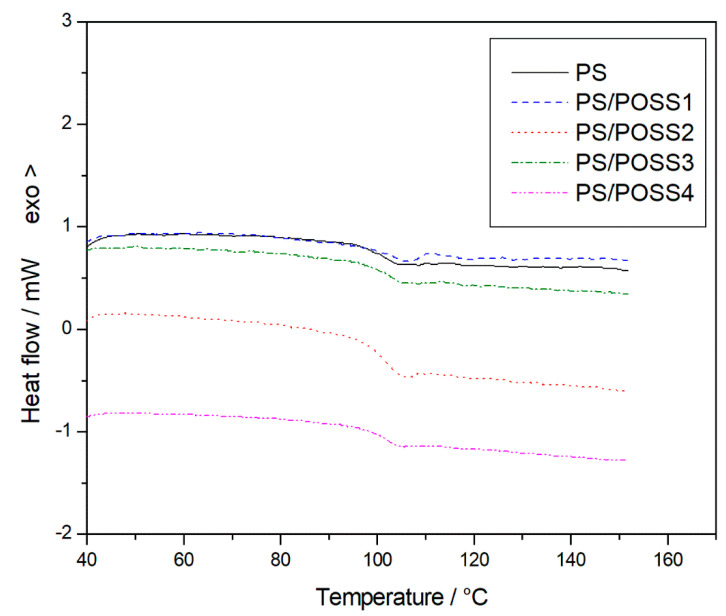
Differential Scanning Calorimetry curves for PS and prepared nanocomposites.

We thus proceed with the calorimetric characterization of PS and PS/POSSs nanocomposites by carrying out DSC scans from room temperature up to 150 °C (Figure 6). In agreement with our previous studies [38,39], no significant changes in glass transition temperature was observed (Table 2).

Just a slight increase in *T*_g_ was recorded for sample **1**, due to the presence of the isobutyl groups in the POSS used for the reinforcement and thus to a slightly different network topology. As we observed in the past, the presence of reactive or less reactive POSSs during synthesis of the nanocomposite may affect the rate of the polymerization reactions thus altering molecular mass and changing the degree of crosslinking and presumably influencing chain mobility [40].

**Figure 7:**
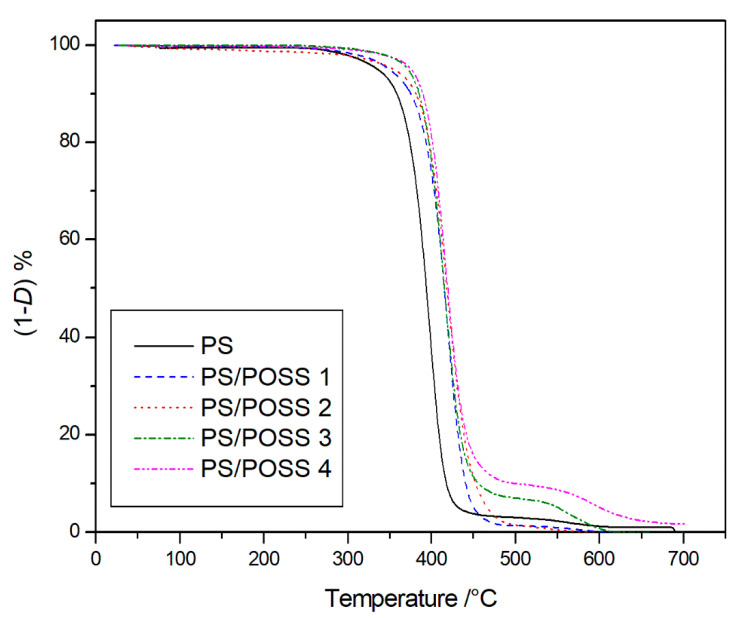
Thermogravimetric curves, in an inert atmosphere, for PS and prepared nanocomposites.

According to our previous works on POSS/polymer reinforced composites and aiming at establishing a comparison among the structures of the POSSs used in this work and those used in the past, we consider the temperature at 5% mass loss (*T*_5%_) and the residue percentage at the end of TGA as parameters to evaluate the thermal performance. Among the different TGA parameters reported in literatures, such as initial decomposition temperature (*T*_i_), temperature at 10% mass loss (*T*_10%_), half decomposition temperature (*T*_hd_), peak temperature (*T*_p_), *T*_5%_ appears us more reliable than the other ones, because it represents a fair value of mass loss whereby a material can begin to be considered degraded and it is not depending on the slope of TG curves. The samples degradations were thus carried out starting from room temperature up to 700 °C, at 10 °C min^−1^, considering that this value is that more employed in literature and, again, allow us the comparison with the previously carried out TG degradations. We started with the degradation in an inert environment, whose TG curves are reported in Figure 7 showing a higher resistance to the thermal degradation for the nanocomposites 2, 3, and 4 with respect to pristine PS.

**Figure 8:**
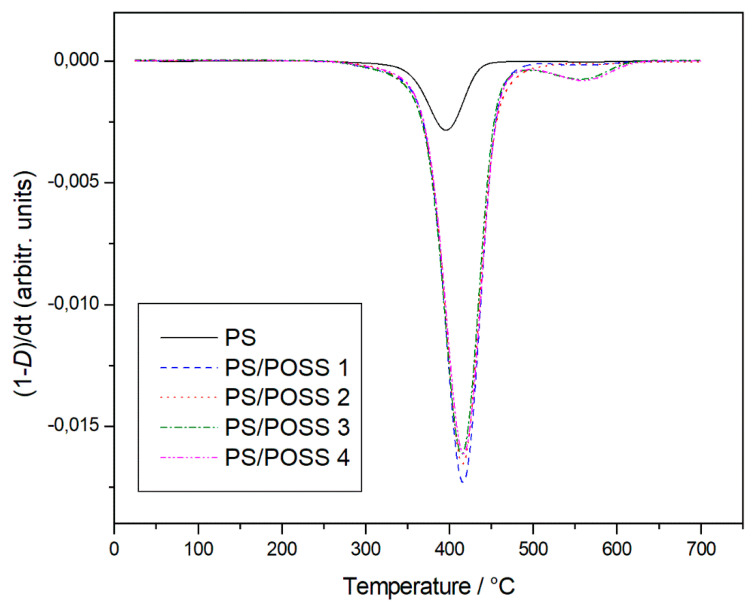
Differential thermogravimetric curves, in an inert atmosphere, for PS and prepared nanocomposites.

The neat polymer and samples **1** and **2** degraded completely in a single stage, whilst the PS composites with three-cage POSS showed a main degradation step, at a temperature slightly higher than that of the single-cage POSS reinforced composites, and a secondary stage of degradation (Figure 8) before completely degrading with sample **3**, and sample **4** showing very little residue. 

The thermogravimetric analysis of the POSS molecules alone has been carried out in a previous study [28]. Considering that the degradations of the POSS molecules alone, in the same investigated Temperature range, led to an amount of the residue at 700 °C ranging from about 30–50 % (depending of the sample and atmosphere); that the percentage of the POSS molecules in the obtained nanocomposites ranging from 6% to 7%. We expected, for the degradation of the PS/POSS nanocomposites an amount of residue at 700 °C ranging from 1 to 4% (depending of the sample and atmosphere) that, considering the experimental error is in line with what was found in the present study (Table 2).

The *T*_5%_ values, recorded for the degradation in inert atmosphere, are reported in Table 2 showing practically the same values for PS and sample **1** and an increase of about 15 °C for sample 2 that becoming more consistent for samples **3** and **4**, about 32 and 35 °C respectively. Returning to Table 2, it is possible to see how the degradation trend for the studied samples in oxidative environment remains exactly the same.

**Figure 9:**
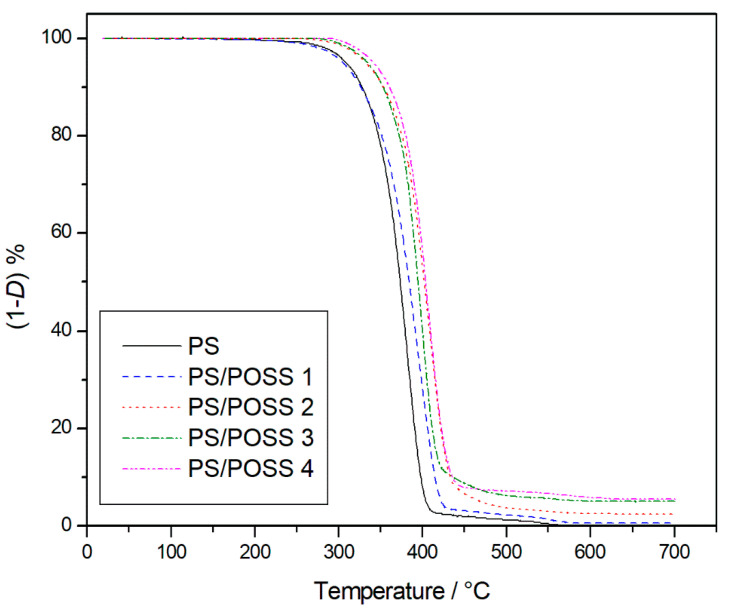
Thermogravimetric curves, in an oxidative atmosphere, for PS and prepared nanocomposites.

Like in inert environment sample **4** showed the higher T_5%_ value, with about the same increment, 31 °C, and also in this case sample **2** showed the same resistance to thermal degradation of neat PS. A difference in oxidative atmosphere was that the degradations of all the nanocomposites showed the presence of a stable residue at 700 °C, whose values increase in the same order observed for the *T*_5%_ values. A very small residue was recorded for polystyrene reinforced with single-cage POSSs with isobutyl groups, which grew for the composite where POSSs were functionalized with cyclopentyl groups. As was to be expected, the residue was maximum when the three-cage POSSs were used in the polymer matrix. The increase of the residue found at 700 °C for the degradations in static air of the samples **3** and **4** corresponded with the disappearance of the second degradation stage, thus all the analysed samples degraded in a single stage in an oxidative atmosphere (Figure 9).

**Figure 10:**
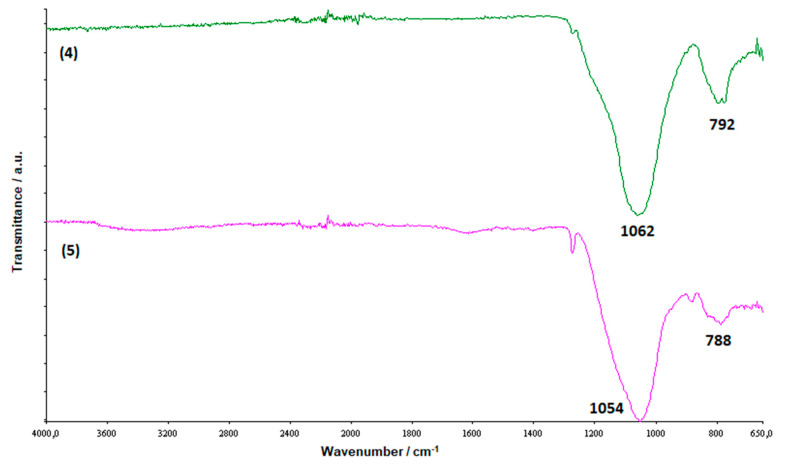
FTIR spectra of the residues at 700 °C, in static air atmosphere, for the samples **4** and **5**.

In order to gain information about the nature of this solid residue, we subjected it to FT-IR analysis that attested its silicic nature (Figure 10), showing the classical bands associated with the presence of silica [41,42].

**Figure 11:**
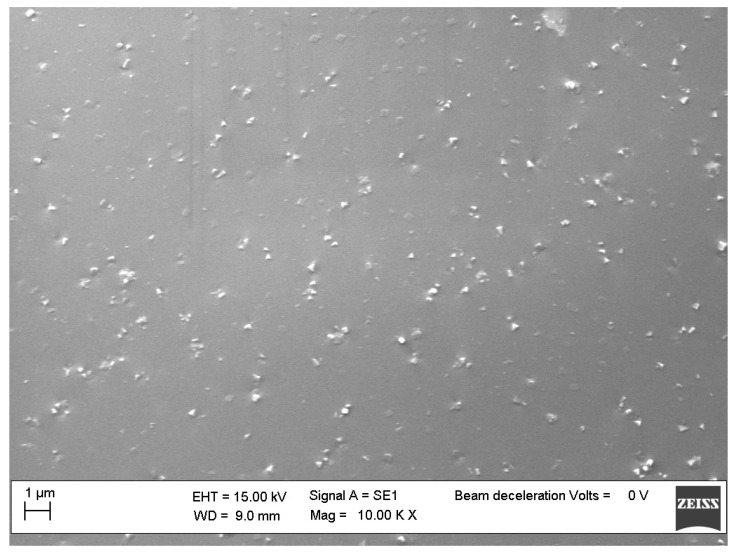
SEM micrographs of sample **4**.

**Figure 12:**
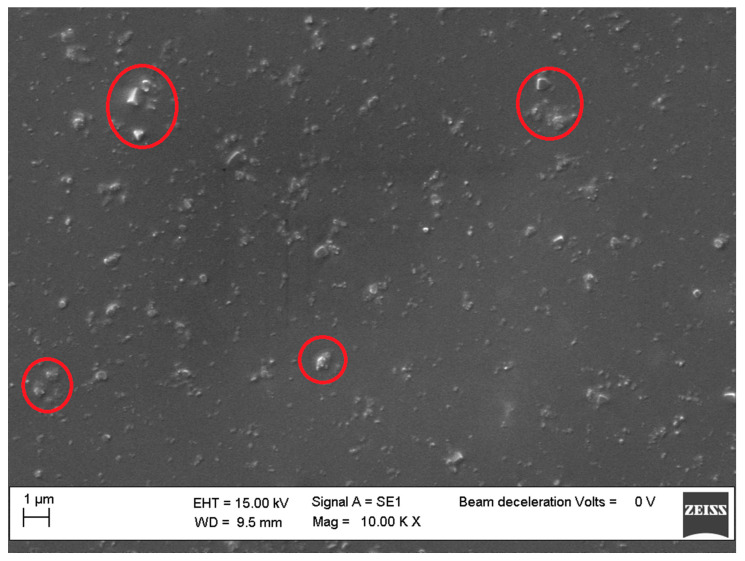
SEM micrographs of sample **1**.

We attributed the better thermal performance of the nanocomposites reinforced with the three-cages POSS molecules to the reduced possibility of aggregates for these bulky nanomolecules compared to those with a single cage. Moreover, their asymmetric structure certainly contributes to further reducing self-aggregation, thus leading to their good dispersion in the polymer matrix, as shown by the SEM images of sample 4 (Figure 11) with respect to a nanocomposite reinforced with a sinlge-cage POSS, showing more aggregation areas (Figure 12).

## 3. Experimental

### 3.1. Materials

Tetrahydrofuran (THF) has been acquired from Aldrich Co. (St. Gallen, Switzerland) and before use was distilled over a Na-benzophenone mixture. Trichloroisobutyllsilane, trichlorocyclopentylsilane and p-tolyltrichlorosilane have been acquired from Aldrich Co. (St. Gallen, Switzerland) and used as received. Trisilanol isobutyl POSS has been acquired from Hybrid Plastics co. (Hattiesburg, MS, USA) and used as received. The cyclopentyl trisilanol (c C_5_H_9_)_7_–Si_7_O_9_ (OH)_3_ was prepared following the reports from literature [43,44]. Methanol has been acquired from Aldrich Co. (St. Gallen, Switzerland) and used as received. Toluene (Aldrich Co., St. Gallen, Switzerland) was stirred over calcium hydride for 24 h and distilled in a nitrogen flow before using. Styrene has been acquired from Aldrich Co. (St. Gallen, Switzerland) and purified in an inhibitor removal column. 2,2-azobis (isobutyronitrile) (AIBN), has been acquired from Aldrich Co. (St. Gallen, Switzerland) and was re-crystallised twice from dry ethanol at temperatures less than 40 °C and in a dark light condition before using. All the reactions were performed under an atmosphere of dry nitrogen. 

### 3.2. POSSs Synthesis 

Octaisobutyl and octacyclopentyl POSS (Table 1) were synthesized by corner capping reaction of isobutyl and cyclopentyl trisilanol [45,46] with trichlorocyclopentylsilane. Both trisilanols and triethylamine were dissolved in dry THF and cooled in two ice baths. The corresponding trichlorosilanes (isobutyl- and cyclopentyl-) were dissolved in dry THF and added dropwise under stirring. The two mixtures were carried out at room temperature and stirred overnight. After filtration of the obtained suspension, the organic filtrate was reduced under vacuum and the resulting slurry was dissolved in a minimum amount of THF and poured into stirred methanol (five-folds). After filtration and drying under reduced pressure, the product (a white solid) was crystallized from a toluene/acetonitrile mixture.

Subsequently, 4-methyl phenyl (trioxyisobutyl POSS) silane [(C_4_H_9_)_7_Si_8_O_12_ -O]_3_ -Si- ArCH_3_ and 4-methyl phenyl (trioxycyclopentyl POSS) silane [(C_5_H_9_)_7_Si_8_O_12_ -O]_3_ -Si- ArCH_3_ (Table 1) were synthesized starting from isobutyl and cyclopentyl POSS mono-ol, which in turn have been prepared from isobutyl and cyclopentyl POSS chloride, respectively.

Trisilanol isobutyl POSS was dissolved in dry THF at 0–5 °C and added with SiCl_4_. The obtained solution was stirred and added with Et_3_N. After stirring overnight at room temperature, Et_3_NHCl was removed by filtration and the filtrate was evaporated to dryness. The resulting solid was dissolved in THF and dry acetonitrile was added to precipitate isobutyl POSS chloride [(C_4_H_9_)_7_Si_8_O_12_]-Cl which was collected and dried. Starting from cyclopentyl trisilanol, the same procedure was followed for obtaining cyclopentyl POSS chloride. The so obtained POSS chloride were used in the preparation of the corresponding POSS mono-ol. A suspension of isobutyl POSS chloride and a mixture 2:1 of THF/H_2_O was refluxed for two days and then rotoevapored. The obtained white solid was dried and crystallized from toluene/acetonitrile thus obtaining Isobutyl POSS mono-ol [(C_4_H_9_)_7_Si_8_O_12_]-OH. Starting from cyclopentyl POSS chloride the same procedure was followed for obtaining white crystals of Cyclopentyl POSS mono-ol [(c-C_5_H_9_)_7_Si_8_O_12_]-OH. Once synthesized isobutyl and cyclopentyl POSS mono-ol, they were, separately and under a nitrogen atmosphere, dissolved in dry THF and added, under stirring, with p-tolyl trichloro silane. The clear solution was cooled at 0–5 °C in an ice bath, added with Et_3_N, and stirred maintaining the temperature at 0–5 °C for 24 h. Et_3_NHCl was removed by filtration and the filtrate was evaporated to dryness obtaining a solid that was then crystallized from a THF/MeCN mixture. White crystals of 4-methyl phenyl (trioxyisobutyl POSS) silane and 4-methyl phenyl (trioxycyclopentyl POSS) silane were thus obtained. ^1^H MNR characterization of the obtained POSS molecules are reported in our previous study [28].

### 3.3. POSSs/PS Nanocomposites Synthesis 

An in situ polymerization were carried out with a 5% (*w*/*w*) POSS/styrene mixture in toluene for obtaining the desired nanocomposites. Each POSS and styrene monomer were dissolved in toluene and AIBN radical initiator. The so mixed solution was frozen in a liquid nitrogen bath, degassed with a vacuum pump, and then thawed. After repeating this operation three times, the tube, sealed under vacuum, was heated at 70 °C for 24 h under stirring. At the obtained solution was added an excess of methanol thus leading to the precipitation of the nanocomposite, which was collected by filtration and dried under vacuum at 40 °C. The same polymerization procedure was used to prepare neat PS. The obtained yields were 77.4%, 78.3%, 76.6%, and 83.2%, respectively for sample **1**, **2**, **3,** and **4**.

### 3.4. FTIR Analysis

Fourier transform IR (FTIR) analysis were performed in a spectrometer by Perkin Elmer (Spectrum 100, Waltham, MA, US) to verify the presence of POSSs in the synthesized nanocomposites an to investigate the nature of the solid residue obtained by the TGA. Investigations wer performed at r.t. from 4000 to 650 cm^−1^ with a resolution of 4.0 cm^−1^. A universal ATR sampling accessory was used for measurements, which were made directly on the samples without further treatment. 

### 3.5. ^1^H NMR Spectroscopy

^1^H NMR spectra were recorded on a Varian Unity Inova spectrometer (^1^H 500 MHz, Varian, Palo Alto, CA, USA). The solvent used for performing measurements was CDCl_3_ whilst tetramethylsilane (TMS) was used as internal standard. The ^1^H NMR data found for the various samples investigated allowed the calculation of the actual POSS percentage in the nanocomposites by taking into account the ratio in the ^1^H NMR spectra among the POSS aliphatic hydrogens and the aromatic hydrogens of PS for compounds **1**–**2** and among the POSS aromatic hydrogens and the PS ones for samples **3**–**4**.

### 3.6. Differential Scanning Calorimetry (DSC)

A calorimeter Shimadzu DSC-60 (Shimadzu, Kyoto, Japan) was used to evaluate the glass transition temperature (*T*_g_) of the prepared nanocomposites. The apparatus was calibrated in temperature and enthalpy using indium, tin and zinc standard materials. About 6 × 10^−3^ g of the prepared compound was placed in punched aluminium crucibles, and subject to a heating rate of 10 °C∙min^−1^ from room temperature up to 150 °C in static air atmosphere.

### 3.7. Thermogravimetric Analysis (TGA)

The thermal properties of PS and corresponding POSSs reinforced nanocomposites were evaluated by thermogravimetric analysis in a Mettler Instrument (TGA 1 Star System, Greifensee, Swizterland). Preliminarily to the measurements the equipment was calibrated following the procedure suggested by the company and used by us in the past [47], which is based on the change of magnetic properties of three metal samples (Isatherm, Nickel-alloy and Trafoperm 86) at their Curie points (148, 355 and 750 °C, respectively). As widely reported in literature [48], often TGA analyzers present an error in the mass determination because the reduction of the buoyancy force on increasing temperature. The International Confederation for Thermal Analysis and Calorimetry (ICTAC) Kinetics Committee recommend carrying out, before the measure, a blank scan with an empty pan, that will then be subtracted from the sample scans, so as to obtain corrected degradation thermogravimetric (TG) curves. An amount of about 5 × 10^−3^ g of sample was put into an open alumina crucible and heated from room temperature up to 700 °C, by using a scanning rate of 10 °C∙min^−1^. The degradations were carried out in flowing nitrogen (0.06 L∙min^−1^) and static air atmosphere. TG data were plotted as percentage of undegraded sample, (1-*D*)% against temperature, where *D* = (Wo − W)/Wo, and Wo and W were the masses at the starting point and during scanning.

### 3.8. Scanning Electron Microscopy (SEM)

The nanocomposites morphology was studied by SEM (ZEISS EVO MA 15, EVO-ZEISS, Cambridge, UK). All of the samples were gold-sputtered up to a thickness of 20 nm by means of an Emitech K-550 sputter coater (Ashford Kent, UK). In order to perform the analysis, an accelerating voltage of 15,000 KV was used. All the samples were treated overnight in an oven at 120 °C to remove any traces of toluene. Scans were carried out at different magnifications ranging from 500× to 25,000×.

## 4. Conclusions

To the best of our knowledge, for the first time three-cages POSS molecules, previously synthesized by us in our laboratory, were used to prepare Polystyrene nanocomposites by in situ polymerization. FT-IR and ^1^H NMR investigations showed the successful outcome of the synthesis both from a qualitative view point (presence of the different POSS molecules in the polymer) and from a quantitative one (POSSs content in the polystyrene), thus validating a well-established preparation methodology. Calorimetric investigations showed no significant changes in glass transition temperature, thus demonstrating no changes in the degree of crosslinking and chain mobility. In the comparison with the classic, and widely used single-cage POSS these novel three-cage POSS molecules showed an increase in the matrix reinforcement ability with a prevalence, in agreement with the literature and with our previous results on different POSSs, of the replaced cyclopentyl substituted compared to the isobutyl ones. Furthermore, we recorded a considerable increase of T_5%_ values in comparison to PS nanocomposite reinforced with montmorillonite organically modified [7]. TGA analyses showed a better resistance to the thermal degradation when carried out in an inert environment, despite the oxidative degradations showed the presence of a solid residue at 700 °C, whose silicic nature was verified by FT-IR analysis. 

## Figures and Tables

**Table 1:** Different POSSs structure used for reinforcing the studied nanocomposites.

POSS used in sample **1**	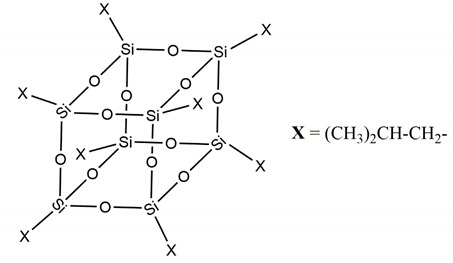
POSS used in sample **2**	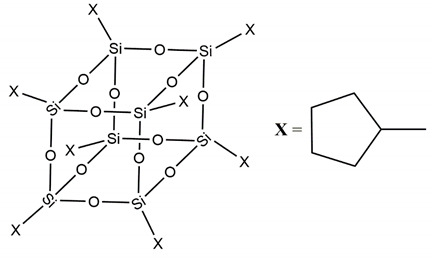
POSS used in sample **3**	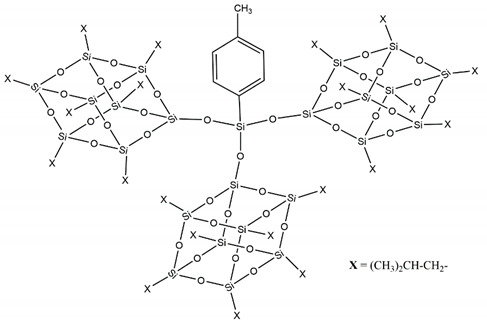
POSS used in sample **4**	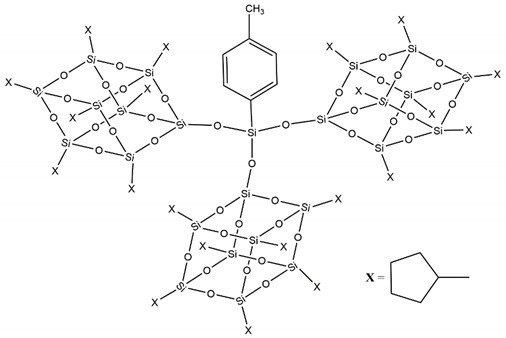

**Table 2:** Glass Transition Temperatures (*T*_g_), Temperatures at 5% mass loss (*T*_5%_) and residue % at 700 °C or PS and synthesized POSS/PS nanocomposites in flowing nitrogen and in static air atmosphere.

Air Static Atmosphere				Nitrogen Flow	
Samples	*T*_g_/°C	*T*_5%_/°C	Residue/%	*T*_5%_/°C	Residue/%
**PS**	100.8	309.2	0	341.1	0
**1**	101.0	308.1	0.62	340.2	0
**2**	101.9	332.8	2.45	355.5	0
**3**	101.5	334.7	5.60	372.3	0
**4**	101.8	341.3	5.11	375.7	1.78
